# Testing the WHO responsiveness concept in the Iranian mental healthcare system: a qualitative study of service users

**DOI:** 10.1186/1472-6963-11-325

**Published:** 2011-11-25

**Authors:** Ameneh Setareh Forouzan, Mehdi Ghazinour, Masoumeh Dejman, Hassan Rafeiey, Miguel San Sebastian

**Affiliations:** 1 Social Determinants of Health Research Centre, University of Social Welfare and Rehabilitation Sciences, Tehran, Iran; 2 Umeå International School of Public Health, Department of Public Health and Clinical Medicine, Umeå University, Umeå, Sweden; 3 Department of Social Work, Umeå University, Umeå, Sweden; 4 Social Determinants of Health Research Centre, University of Social Welfare and Rehabilitation Sciences, Tehran, Iran; 5 Department of Social Welfare Management, University of Social Welfare and Rehabilitation Sciences, Tehran, Iran

## Abstract

**Background:**

Individuals' experience of interacting with the healthcare system has significant impact on their overall health and well-being. To relate patients' experiences to a common set of standards, the World Health Organization (WHO) developed the concept of health system responsiveness. This study aimed to assess if the WHO responsiveness concept reflected the non-medical expectations of mental healthcare users in Teheran.

**Methods:**

In this qualitative study, four mixed focus group discussions were formed, comprising 53 mental health service users in Tehran, Iran, in 2010. Content analysis was performed for data analysis. Responses were examined in relation to the eight domains of the WHO's responsiveness model.

**Results:**

There were many commonalities between the findings of this study and the eight domains of the WHO responsiveness model, although some variations were found. Effective care was a new domain generated from our findings. In addition, the domain of prompt attention was included in two new labelled domains: attention and access to care. Participants could not differentiate autonomy from choice of healthcare provider, believing that free choice is part of autonomy. Therefore these domains were unified under the name of autonomy. The domains of quality of basic amenities, access to social support, dignity and confidentiality were considered to be important for the responsiveness concept. Some differences regarding how these domains should be defined were observed, however.

**Conclusions:**

The results showed that the concept of responsiveness developed by the WHO is applicable to mental health services in Iran. These findings might help policy-makers' better understanding of what is useful for the improvement of mental health services.

## Background

Individuals' experience when interacting with the healthcare system has a significant impact on their overall health and well-being [1]. This includes the manner and environment in which people are treated when they seek health care. To relate patients' experiences to a common set of standards, the World Health Organization (WHO) developed the concept of health system responsiveness [2]. This concept, based on the Donabedian framework of quality of care, reflects how well the healthcare system responds to the population's expectations regarding non-health aspects of health care [3]. Responsiveness is also one of the three fundamental objectives of a health system, together with good health and fair finance [2].

The development of the responsiveness conceptual framework and the methodology for measuring it arose from a broad literature review of the quality of care [4]. The review focused on the question of what, apart from improving health, was valued by healthcare system users. Public health practitioners, ethicists and healthcare professionals contributed to its development [5]. The outcome of this process ended with eight domains for measuring responsiveness which are shown in Table 1[6]. These domains are applicable to all types of health care [7].

**Table 1:** Definition of responsiveness domains by WHO

Domain	Definition
Dignity	being shown respect having physical examinations conducted in privacy
Autonomy	being involved in deciding on your care or treatment if you want to having providers ask your permission before starting treatment or tests
Confidentiality	having conversations with healthcare providers where other people cannot overhear having your medical history kept confidential
Clear communication	having healthcare providers listen to you carefully having healthcare providers explain things so you can understand giving patients and family time to ask healthcare providers questions
Prompt attention	getting care as soon as wanted having short waiting times for tests
Access to social support networks	being able to have family and friends bring personally preferred foods, and other things to the hospital during the hospital stay being able to observe social and religious practices during hospital stay access to newspapers and TV interacting with family and friends during hospital stay
Choice of healthcare provider	being able to see a healthcare provider you are happy with being able to choose the institution to provide your health care
Quality of basic amenities	having enough space, seating and fresh air in the waiting room or wards, having a clean facility

Source: Valentine 2003

Good responsiveness is especially pertinent for the mental healthcare system. It is expected that responsiveness will impact positively on mental health outcomes, since it will decrease the relapse rate and enhance patient compliance [8]. Owing to the specific vulnerability of mental health patients because of the chronicity of some mental disorders, the characteristics of some treatments (coercive treatment and drug side-effects), as well as the stigma attached to mental health care, responsiveness is even more relevant in this field [9]. Although conceptually the indicators of responsiveness seem to be reasonable, asking health service users for their opinions can help to make the domains even more relevant. In 2008, the WHO asked countries to review their health policies in the light of the Primary Health Care approach (PHC), where social participation is a key component [10]. Social participation in health is considered a determinant tool in increasing health systems' responsiveness. When patients are given a voice, it becomes possible for them to influence change and facilitate improvement in the system. Understanding populations' perceptions of quality of care is also critical for developing measures to increase the utilization of primary healthcare services. Nevertheless, the application of the concept of responsiveness to the mental healthcare system has been limited. To our knowledge only two studies from Germany have tested its applicability [9,11]. Given the importance that different socioeconomic, cultural and religious contexts play in mental health, however, it is necessary to explore the domains in particular local settings [12].

The Islamic Republic of Iran is one of the most populous countries in the Middle East (67.5 million, of whom 44.8 million (66%) live in urban areas). The literacy rate is 83.5% for males and 69.9% for females [13]. The official language is Farsi, and nearly all ethnic groups are formally educated in Farsi [14]. Although the country is confronted with relatively high levels of income inequality, poverty and unemployment, it is advanced in terms of health and education. The national health insurance system covers more than 90% of the population [15], although some payments, such as fees for non-medical psychiatric treatments, are not covered by the health insurance.

Iran is undergoing a demographic and epidemiological transition which will have a significant effect on the evolution of the patterns of morbidity and mortality in the future [16]. Mental health disorders have become more prevalent in recent years; according to a national survey in 2001, the point prevalence of mental disorders was estimated to be around 22% [17].

Since the late 1980s, Iran has pursued full integration of mental health care into the national primary care structure [18]. The aim was to establish a hierarchical, pyramid-like referral system [19], which would improve physical access to mental health services. Reports have pointed to the limited integration in urban areas, showing very poor case detection [20]. The urban coverage of the programme has also been lower when compared with rural areas (21.7% and 82.8%, respectively, in 2004) [19]. Despite the fact that the majority of mental health professionals, including all psychiatrists, are currently working in large cities, service delivery is disorganized and most services are hospital- and clinic-based [21]. The majority of the users are treated in medical-oriented outpatient clinics and mental hospitals (948 and 130.4 patients per 100,000 people, respectively). The rate of users consulting day treatment facilities and community residential facilities, where extra services such as rehabilitation or counselling are offered, is low (2.8 and 6.0 per 100,000 people, respectively) [13]. There are a total of 33 mental hospitals, with 7.9 beds per 100,000 people, which is low compared with other countries in the Middle East region: 9.7 beds per 100,000 persons in Qatar, and 14 beds per 100,000 in the United Arab Emirates [13,22].

Although there have been some efforts to evaluate the mental health system [23], less attention has been paid to the assessment of the quality of care, particularly in large cities. In a rapidly changing society such as the Islamic Republic of Iran, rapid urbanization and epidemiologic transition require a shift in attention towards urban health needs [13]. Being able to evaluate the quality of mental care based on objective standards could be valuable in terms of finding gaps and areas for future attention. The WHO instrument for assessing responsiveness could be a useful tool in this regard. This study aimed to assess if the WHO responsiveness concept reflected the non-medical expectations of mental healthcare users in Teheran. In order to begin to understand patients' experiences the study, based on a qualitative approach, focused on two main questions:

- What are the non-medical qualities regarding mental healthcare services that are considered relevant by mental healthcare users?

- Could the WHO's responsiveness concept with its eight domains be expanded appropriately to reflect the non-medical expectations of mental healthcare users?

## Methods

### Setting

The current study took place in Tehran, the Iranian capital, which has a population of approximately 12 million. There are five mental health hospitals offering comprehensive mental health services under the supervision of public medical universities. The city is divided into five main areas (North, South, East, West and Central), and each hospital covers one part. Referrals from the different divisions and from other cities are common. These hospitals provide inpatient as well as outpatient services. University mental health hospitals have the highest referral level and all of their facilities are integrated with the mental health outpatient facilities. Patients can choose freely where they want to be treated. Medical costs are covered by social health insurance [13]. For patients without insurance coverage, the fee for services is lower than in the private sector.

### Participants

Four mixed focus groups, comprised of 53 male and female mental health service users, were formed [24]. The inclusion criteria for participation were: (1) being adult (18 to 65 years old); (2) having at least one year's experience of using mental healthcare services (outpatient as well as inpatient); (3) not being acutely ill; (4) being cognitively capable of participating in a group discussion. The type of participants' mental disorders was not treated as an inclusion criterion since current diagnoses of patients do not have any relationship with experiences of mental health services [25]. Participants were recruited from outpatient service facilities, affiliated to one of the public medical universities, with the assistance of mental health service providers. Two groups came from an outpatient centre, one from a daycare facility and one from a non-governmental mental health rehabilitation centre. The reasons for choosing these centres were accessibility and a previous history of collaboration with the research team. The sample centres were representative of types of services, as they had a previous history of using outpatient as well as inpatient services. The group discussions were carried out at one of the outpatient mental health facilities, located in the South division of Tehran. Data collection started in June 2010 and ended in August 2010.

### Data collection

Focus group discussions were used because of their appropriateness for exploring the applicability of responsiveness in the mental healthcare system [9]. The discussions started by presenting the aim of the study. The participants were also informed during the discussion about confidentiality and their right to withdraw from the study at any time. The nature and purpose of the study were explained to each participant before they gave their consent, which was confirmed by a signature. Permission to audiotape the interview session was sought orally prior to the interviews. At the beginning, participants were asked and encouraged to talk openly about what they expected from an ideal mental health centre. The moderator then gave the group some information about the responsiveness concept based on the WHO definition. After this, the model was discussed domain by domain, and participants were asked to discuss the applicability of each domain regarding mental health services. Probes were used to confirm concepts mentioned and to explore new areas. Following the discussion, a short questionnaire for assessing demographic data, as well as life-time contacts with mental healthcare services, was filled in by the participants. The first author moderated all the discussions. Each focus group lasted 1.45 to 2.0 hours, and ended when no new issues seemed to arise. The discussions, in Farsi, were audiotaped and then transcribed. The study protocol was approved by the University of Social Welfare and Rehabilitation Sciences Research and Ethical Council.

### Data analysis

Content analysis was performed for data analysis [26]. The transcribed interviews were analysed manually. Three researchers coded the transcripts independently. The transcripts were read with the intention of deriving 'meaning units' (covering words, phrases, and/or paragraphs) [27]. The coding scheme was derived theoretically, according to the concept of the responsiveness and its domains. Citations were assigned deductively to eight categories which represented the domains of the WHO's responsiveness concept. On the other hand, the phrases and codes were identified from transcripts, providing the basis for generating new categories or subcategories, as well as modifying the categories developed by induction. The inductive subcategories were sorted into meaningful clusters within the theoretical categories [28]. Citations were only coded once with the category best representing the focus of the statement. The coding was synchronized between the researchers through two discussion sessions, each taking four hours, to ensure the credibility of findings [24]. The results were then discussed with two senior researchers to strengthen plausibility.

## Results

### Participants' characteristics

Table 2 shows the characteristics of study participants as well as their experience regarding attendance at different mental health services. The mean age of participants was 34.4. The youngest participant was 19, the eldest 78. Gender distribution was almost identical in both groups. About one-third of participants was unemployed, about 90% of them had access to insurance services and 37% had a previous history of hospitalization.

**Table 2:** Characteristics of study participants

Variables	Frequency	%
Sex		
Female	25	46.2
Male	29	53.7

Education		
Below diploma	4	7.4
High school diploma	36	66.6
Above high school diploma	14	25.9

Employment status		
Unemployed	17	31.4
Housekeeper	13	24.0
Student	3	5.5
Employed	15	27.7
Domestic employment	3	5.5
Missing	3	5.5

Insurance coverage		
No	5	9.2
Yes	44	81.4
Missing	5	9.2

Type of mental health care		
Outpatient clinic	29	53.7
Both outpatient and inpatient services	20	37.0
Missing	5	9.2

### Content analysis of discussions

The responses were examined in relation to the eight domains of the WHO's responsiveness model. Subcategories were generated deductively and the quotations that did not fit into any of the WHO domains were categorized inductively. A new domain, effective care, was generated. It is important to mention that participants could not differentiate autonomy from choice of healthcare provider. They believed that free choice is part of autonomy. Therefore, these domains were unified under the name 'autonomy'. The domain of *prompt attention *was included in *attention and access to care *on the basis of the declared expectation of the participants. Table 3 shows the newly formed and expanded categories and subcategories related to each domain, as explained by participants.

**Table 3:** Results of the categories and related subcategories found in the analysis

**Category**	**Sub-categories**	**Definition**
Attention**	Close relationship	Close and affable dialogue between mental health workers and patients
	Insightful listening	To attend to and to respond with deep understanding to the patients
	Enough time	Having enough time to ask questions about mental health problem or treatment
	Thoughtful care	Proactive and careful follow-up of the process of treatment by service providers
	Empathy	Mental healthcare providers show they understand how patients feel about their problem
Dignity	Respectful care	Showing respect when treating patients
	Non-stigmatizing treatment	Not being stigmatized when dealing with service providers
	Taking patients seriously	Patient problems and complaints are taken seriously
	Maintaining individuality	To recognize patients' individual needs and characteristics
Clear communication	Informative counselling	To provide patients with understandable information about their problem
	Comprehensibility of information	To provide information about patient problems in a comprehensible manner
Autonomy	Choice of care	Services and providers can be chosen freely
	Participation in care process	To be able to participate in therapeutic decisions and processes
	Feeling equal power	Patient/provider relationship at the same level
Effective care*	Practicality	To provide practical advice in congruence with patient norms and values
	Continuous care	Continuity of care across services and sectors, to provide care by same familiar person
	Appropriate use of resources	To provide services commensurate with costs such as time and money
Access to care**	Convenient access to acceptable care	Acceptable care provided as soon as needed by patient
Confidentiality	No subcategories found	To handle patients' information confidentially
Quality of basic amenities	No subcategories found	To be treated in clean, informal and friendly places
Access to social support	No subcategories found	Contact with family, friends and peers is promoted

* New categories

** New labelled category

#### Attention

The majority of statements were related to this domain. Participants discussed the quality of the interaction with the care providers which was beyond the original concept of prompt attention. A warm and sincere approach was expected by almost all participants. Statements related to this domain were categorized as: close relationship, insightful listening, enough time, empathy and thoughtful care.

• Close relationship

Participants revealed that they expected a close and affable approach, based on a sincere dialogue, from the mental health providers: '... because of the nature of our illness they should be kind and treat us with kindness; this is more than what we expect from other medical specialties... '. A formal approach was undesirable for the majority of the respondents: 'When they talk to me formally I feel uncomfortable, therefore I cannot talk about my deep feelings and problems'. Spending enough time chatting before starting the consultation was an example of the close relationship mentioned by respondents. Several pointed out that this warming-up time was useful for reducing stress.

• Insightful listening

Insightful listening to the patient's needs and complaints was considered as part of attention. The majority of respondents said that they could tell if they were being listened to insightfully or not, mainly through non-verbal communication: 'You know, when he did not look at my eyes and checked the clock repeatedly during the interview, I realized that he did not care...'.

• Enough time

More than half of the participants said that they expected to be allocated more time with the mental health workers: '... I am sure that if he spent more time on the consultation, I would have more important things to disclose'. Time limitation was also considered as a source of distress: 'I am always worried about the time. This makes me nervous. I cannot concentrate well then'.

• Thoughtful care

Respondents expected that the mental health staff would follow up the process of treatment carefully and support them actively during this process. Proactive follow-up was also included under this issue: 'Owing to the drug side-effects I forget things; this includes the time of medical visits, so I expect that they will remind me...' and 'We need direction. A month before New Year's Eve, I was isolated. Therefore, I stopped going there but nobody called me to ask why I did not come ...'.

• Empathy

According to participants' statements, an important component of attention was empathy and deep understanding. Empathy was important for a trusting relationship: 'When I see that he understands me well, then I can trust him and disclose my thoughts and feelings'. There were several participants who believed that, occasionally, empathy could be as useful as medication. There were some negative comments that illustrate how participants do not want to be treated: 'They don't really understand us. I feel it from their non-verbal actions.'

#### Dignity

This domain consisted of four subcategories: respectful care, non-stigmatizing treatment, taking patients seriously and maintaining individuality. Most of the respondents revealed that this is an important issue, especially the lack of respectful care, which was associated with failure to comply with the treatment.

• Respectful care

More than two-thirds of the statements regarding dignity were related to respectful care. This included the expectation of a humanistic approach by the service providers: 'They should treat us like human beings and consider our rights'. Mutual respect was another expectation: 'There should be mutual respect between us; I am an adult just like them even though I am mentally ill'.

A major issue was about the consequences of disrespect: 'I don't take my pills because they gave me a handful of pills. It's very discourteous'. 'When I feel that he does not respect my cultural norms and values, then I decide not to visit him any more'.

• Non-stigmatizing treatment

Not being stigmatized when dealing with service providers was discussed in all groups. A significant number of participants stated that they had a positive experience of not being stigmatized when referring to the mental health services.

• Taking patients seriously

Participants revealed that their complaints were not taken seriously by some of the service providers: 'I was depressed but not stupid when I claimed that I could not tolerate the drug side-effects, but they did not pay attention to me'. 'When they visit us they just focus on our mental symptoms, they don't understand that when we talk, we need to be taken seriously'.

• Maintaining individuality

More than one-third of the statements regarding dignity were categorized under maintaining individuality: 'They should classify us based on our strengths and weaknesses; they should recognize our needs one by one'. Not mixing up severely ill patients with others was also highlighted during the discussions.

#### Clear communication

Statements related to this domain were categorized as informative counselling and comprehensibility of information.

• Informative counselling

The majority of participants in all groups expected that their therapists would provide them with detailed information about their disorder. In addition, they expected this information to be in plain language and not couched in professional terms: 'I need to know exactly what is wrong with me; sometimes the professional words they use make me anxious and more confused'. 'Just repeating that I have to take pills is not enough; I want to know exactly what I should do. For example, when you have diabetes it is not enough to tell you to observe your diet; they should tell you exactly how'.

• Comprehensibility of information

Some participants claimed that because of issues such as time limitation, therapists refuse to give them detailed information. Furthermore, when they did not get enough information and their questions were not replied to accurately they felt that they were unvalued. '... I still don't even know the name of my illness ... he thought I was stupid and I didn't understand him...'.

#### Autonomy

The statements related to this domain were categorized as: choice of care, participation in care process and feeling equal power.

• Choice of care

Participants expected to have the freedom of choice when they were not psychotic and when they did not pose a danger to themselves or others. Several highlighted the need to be satisfied with the process of therapy and, during this process, to feel free to change care centre or provider. Their concern, however, was about barriers to free selection. They explained that this freedom of choice is not usually guaranteed. Choosing the gender of care provider was also clearly stated as being fundamental, especially by female participants: 'There are some topics that I cannot discuss with a male therapist. I feel ashamed when I have to do this....'.

• Participation in the care process

Although most participants expected to play a significant role in their care process, all of them agreed that dangerous patients and those who were addicted to substances should be considered as exceptions: 'Addicted patients are out of their minds. They'll do anything to get drugs. Of course they cannot take part in their treatment before detoxification....'. Several pointed out that having information about their disorder is a prerequisite for participation.

• Feeling equal power

Less than one-third of the statements related to autonomy were categorized as feeling in an equal position or having equal power. This category was cited by respondents as a precondition for participation. 'They think that doctors are the only ones who know everything....Of course we may not be literate but we know what works and what does not....'. 'When they consider us as low class people they don't even ask for our participation...'.

#### Effective care

This category was not included in the WHO model. Statements which were categorized under this domain were the experiences and expectations of participants regarding the care outcome. Practical health advice, continuous care and appropriate use of resources were subcategories generated under this domain.

• Practical advice

Participants expected practical advice, especially in situations where making a decision was difficult for them: 'During the active phase of my illness it is really difficult for me to think rationally. That is why sympathy alone is not enough. I need practical advice then'. Cultural congruence with the recommendations was also considered under this subcategory: 'His advice was no use to me. In fact, I paid for nothing. The norms and values I believe in contradict what he wants me to do'. Several participants mentioned that a good example of practical advice appeared when the healthcare workers acted according to what they said. 'I think they could not do what they recommend to us. That is why I think advice from other patients is much more useful than what the professionals recommend'.

• Continuous care

Some participants revealed that the rotation of mental health staff produces discontinuity of care. This was a source of stress and dissatisfaction. 'Each time I am referred to an outpatient clinic, I meet a new therapist. This makes it very difficult to develop trust'. Another aspect of this subcategory was the existing gap between different mental health services. 'Therapists do not work as a team. Sometimes they even give contradictory advice. This makes me very confused'.

• Appropriate use of resources

Although this domain was mentioned only a few times, the consequences of costly but ineffective services were serious. 'It is not just money that you have to pay for; you spend your time too. That is why when I felt it was not worth it I decided to withdraw'.

#### Access

Access to care was also a new labelled domain and covered the availability of acceptable mental health services when needed. Participants expected short intervals between visits, convenient travel to mental health centres and availability of care in emergency situations. 'Each time I have to come here I feel stressed; that is, because of the long distance, I am afraid I won't arrive on time...'. Convenient access to health professionals and receiving care at short notice becomes more important in critical situations. 'Easy access to the mental health centre is also more important. When we feel desperate we need to get help immediately'.

#### Confidentiality

All groups had very challenging discussions about confidentiality. One of the main concerns was about keeping patients' information confidential. Several participants believed all their information should be kept secret and nobody should know about it except the therapist. They were afraid that if their family knew about their mental disorder, they would humiliate them. Others, however, believed that the therapists had the right to discuss their information with those who might have some influence. 'My therapist should call my husband and talk to him about my problem. He should know what has happened to me'. 'The therapist is authorized to share our information. He is like our parent. He knows what is good for us and to whom he should talk about our illness'.

#### Quality of basic amenities

This domain was important for practically all participants. They expected to be treated in clean and tidy places. The majority of respondents revealed that they have had both positive and negative experiences in this regard. About one-third of them said that this topic was less important than being treated with dignity and receiving attention from care providers. Others insisted that the environment of the mental health centres should be informal and friendly. They believed that the quality of the surroundings could be more influential for them than for non-psychiatric patients: 'Using plants and photos to decorate the rooms makes us feel at home...' and '... we need comfort more than other patients; we are sensitive to stimulants when it is too warm or too noisy, which we cannot tolerate'.

#### Access to social support

In this category participants referred to the need to have contact with family and friends, especially when they are admitted as inpatients, as well as access to peer support: 'Of course, it's good that they come and visit us daily, the food that they bring means a lot, it means they think about us and remember us', '... we need to have free access to family and friends when we are admitted into the hospital: it is intolerable to be locked in wards', '... having group meetings with other patients is very useful, I usually learn more from their advice than from my therapist's recommendations...'.

## Discussion

To our knowledge this is the first study to apply the WHO's concept of responsiveness to the specific subsystem of mental health care in a middle-income country. Though there were many commonalities between the findings of our study and the WHO's concept of responsiveness, some variations were also found. A new domain was conceptualized and some of the existing domains were labelled anew, expanded and integrated.

### The common domains

In accordance with findings from previous studies, quality of basic amenities, access to social support and confidentiality were the most discussed domains [9,12]. The expectations regarding quality of basic amenities were more than the physical characteristics of health centres, as defined in the WHO model. Participants in all groups expected to be treated in a home-like environment. This difference seems to reflect the poor infrastructure and cold environment of many mental healthcare facilities in Iran [21]. When discussing access to social support, respondents expected to have good access to family and friends, as well as peer support. During recent decades, considerable evidence has shown that peer support decreases the relapse rate and improves the social relationships of patients [29,30]. Unfortunately, there is a lack of these types of programmes within the mental health system in Iran [31]. Confidentiality was vividly discussed in all groups, the main concern being about the limit of secrecy. This concern is highly relevant since no clear guidelines exist in the country. Space for discussion by patients, caregivers and professionals should be allowed to clarify the concern. This issue has also been discussed in other studies where caregivers often need certain information to enable them to provide effective support [32].

### The domains expanding the concept

The domains of dignity and clear communication were highlighted as relevant to the concept of responsiveness, but the definition of these domains was more expansive than the WHO description. One possible explanation could be the specific meaning that these two domains might have in the mental health context [33]. Negative attitudes and stigma attached to mental illness have the potential to engender disrespectful care [34] and thus raise negative feelings among service users. Therefore, it is really important to improve the training of mental health staff and try to modify their practice. The nature of emergency psychiatric situations and the shortage of psychiatric services in urban areas in Iran [21] may influence the amount of time spent with a patient, impeding clear and desirable communication with them.

### The integrated domain

In contrast to previous studies, the domain of choice of healthcare provider was integrated into the autonomy one [2,9]. Participants in all four groups agreed that only when they had enough power and information were they able to make choices about their mental health care. Other studies related to mental health services have also confirmed that the domain of autonomy has a specific meaning in the mental health context [11]. Campbell believed that social position in a community of people with a mental illness might be a reflection of the disempowering practices of mental healthcare professionals [35]. Others have attributed this to the medicalized approach to mental health issues, instead of one considering the social context related to this chronic condition [36]. The main concern about this domain, according to participants' explanations, was about the limits of their autonomy and the practical guarantees of support for their autonomy. This could be explained partly as a result of the lack of a mental health act guaranteeing the right to autonomy and freedom of choice for psychiatric patients in Iran. Although a professional team, including law experts, has prepared a first draft of a mental health act, the process of amending and presenting it to Parliament needs greater effort [37].

### The new labelled domains

Quality of attention to patients' needs was treated as a new labelled category, called *attention*. It became very clear from the discussions how the participants wished the interpersonal relationships between healthcare professionals and patients to be conducted. The demand for close relationships, insightful listening, thoughtful care, empathy and dedicated time are distinct warnings for mental health professionals. Complaints in this area may be related to shortage of mental health staff and facilities as well as unbalanced distribution of the services (13, 22).

Access to care was a newly labelled domain, too. Though it was similar to the WHO domain of prompt attention, some differences could be observed. Both availability and acceptability of services were expected by participants. Once a service is physically accessible, it still needs to be acceptable. Although enormous efforts have been made to integrate mental health into the PHC system, a recent study assessing the mental healthcare system in the Islamic Republic of Iran concluded that mental health services are facing important challenges in cities. The availability of only very centralized psychiatric institutions, as well as a lack of mental health funding and staff, might negatively influence access to care [21,23]. Providing acceptable services for patients was the other topic discussed in this domain. There is a great need for a broad picture of how the mental health process, as well as the treatment, is perceived by patients [38]. The delivery of acceptable mental health care to patients is complex and requires strategic planning and flexible resourcing [39]. On the other hand, it is also important to consider changing the negative and false beliefs about mental and neurological illnesses that still exist among the population.

### The newly formed domain

The domain of effective care was a newly generated domain. Continuous care, practical advice and appropriate use of resources were three subcategories of this domain. This concept has also been defined as part of technical and performance quality in other studies working on the overall quality of health care [40]. Considering continuous care as part of the responsiveness concept is also supported by other studies in the field of responsiveness and quality of care [9,41]. Expecting a long-term relationship with care providers is part of the chronic nature of the illness [9]. Lack of coordination within mental health services and with the rest of the healthcare system, particularly in urban areas, might be the main reason behind the patients' complaints [23].

Practical advice and recommendations by care providers was another subcategory related to effective care. It seems that the advice given by the professionals was not found to be very useful by the patients. Practical advice has been reported as important in other studies evaluating mental health service quality and utilization [42]. Studies have shown the importance of reorienting healthcare services to make them practical and to improve professional skills in this regard [43].

The appropriate use of resources, particularly fees and time, was another subcategory referred to by the study participants. Although the majority of the population is covered by health insurance, the fact that some medications and non-medical therapies are not covered might hinder the access of some poor patients to them. Decentralized services as well as community-based services could significantly influence the appropriate utilization of resources and improve the quality of mental health care.

## Conclusions

This study has proved that the concept of responsiveness developed by the WHO is applicable to mental health service users' expectations in Iran. Service users also had additional expectations, however. This implies that a new domain, effective care, needs to be added to the WHO's concept in the Iranian context. In addition, the domain of choice should be integrated into the domain of autonomy, and the domain of prompt attention needs to be changed to a new domain called access to care. Expectations regarding the quality of interaction, which was the major theme of all discussions, needs to be categorized as 'attention'. The domains of dignity and clear communication require expansion compared with the WHO definition.

The results presented could be useful for increasing awareness of how patients conceptualize the concept of responsiveness, and of what they expect when dealing with mental health services. They could well help policy-makers' better understanding of patient expectations and provision of better mental health services. Technical improvement for better responsiveness is important but not sufficient. In addition, some simple and affordable interventions, such as changing the physical environment of mental health centres, can impact positively on patient care.

Since responsiveness in its broad concept reflects the expectations of service users, a further question would be to what extent users' expectations are actually met by the mental healthcare system. To explore this issue, a quantitative survey of responsiveness would be required. It should be emphasized that to operationalize this concept we need to control the overlap that might already exist in the present conceptualization of domains. In addition, it is important to ensure that selected categories are translated into items/questions that are feasible for measurement from the patient's perspective.

## Competing interests

The authors declare that they have no competing interests.

## Authors' contributions

ASF: Conception and designing of the study, data collection, qualitative analysis of data, interpretation of data and drafting the manuscript, critical revision of the content of manuscript. MGH: Planning of designed data collection, analyzing the data, interpretation of data, reading and commenting the manuscript and the revised version. MD: Qualitative analysis of data, interpretation of data and drafting, critical revision of the content of manuscript. HR: Qualitative analysis of data, interpretation of data and drafting, critical revision of the content of manuscript. MSS: Designing of the study, qualitative analysis of data, interpretation of data and drafting, critical revision of the content of manuscript.

All authors read and approved the final manuscript.

## Pre-publication history

The pre-publication history for this paper can be accessed here:

http://www.biomedcentral.com/1472-6963/11/325/prepub
